# Supplementary material for: Mechanisms governing the pioneering and redistribution capabilities of the non-classical pioneer PU.1
Source: Nat Commun. 2020 Jan 21;11:402. doi: 10.1038/s41467-019-13960-2 (PMC6972792; doi:10.1038/s41467-019-13960-2)
Supplement: Supplementary file 7 — Source data [file 41467_2019_13960_MOESM7_ESM.zip › Source_Data/FigureS2/FigureS2B_MotifScanOutput/KmeansCluster7/homerResults/motif18.similar.html]

motif18

## Information for motif18

C
T
G
A
C
G
T
A
G
T
C
A
C
T
A
G
C
G
T
A
T
C
A
G
A
T
C
G
C
G
T
A
C
G
T
A
C
A
T
G
C
G
T
A
C
T
A
G
A
C
T
G
  
Reverse Opposite:  

T
A
G
C
A
G
T
C
A
C
G
T
G
T
A
C
A
C
G
T
A
C
G
T
A
T
G
C
A
G
T
C
C
G
A
T
A
G
T
C
A
C
G
T
A
C
G
T
A
G
C
T
  

|  |  |
| --- | --- |
| p-value: | 1e-63 |
| log p-value: | -1.460e+02 |
| Information Content per bp: | 1.787 |
| Number of Target Sequences with motif | 127.0 |
| Percentage of Target Sequences with motif | 3.96% |
| Number of Background Sequences with motif | 189.3 |
| Percentage of Background Sequences with motif | 0.43% |
| Average Position of motif in Targets | 108.1 +/- 52.5bp |
| Average Position of motif in Background | 114.1 +/- 90.9bp |
| Strand Bias (log2 ratio + to - strand density) | 1.4 |
| Multiplicity (# of sites on avg that occur together) | 1.00 |
| Motif File: | file (matrix) reverse opposite |

### Similar de novo motifs found

|  |  |  |  |  |  |  |  |
| --- | --- | --- | --- | --- | --- | --- | --- |
| Rank | Match Score | Redundant Motif | P-value | log P-value | % of Targets | % of Background | Motif file |
| 1 | 0.853 | G T C A T C G A A T C G C T G A C T A G T C A G T G C A C T G A T A C G C G T A A C T G A C T G G T C A | 1e-60 | -138.335870 | 5.14% | 0.89% | motif file (matrix) |
| 2 | 0.792 | T A C G G T C A T C G A T A C G G C T A C A T G T A C G C G T A C T G A C A T G C G T A C A T G A T C G C T G A | 1e-47 | -108.993338 | 5.11% | 1.15% | motif file (matrix) |
